# Advance Care Planning and Communication Skills Improve after an Interprofessional Team Simulation with Standardized Patients

**DOI:** 10.1089/pmr.2021.0086

**Published:** 2022-08-08

**Authors:** Leah S. Millstein, Paula Rosenblatt, Melissa H. Bellin, Laura Whitney, Steven R. Eveland, Mei Ching Lee, John Allen, Heather L. Mutchie, Todd D. Becker, John Cagle

**Affiliations:** ^1^ Department of Medicine, University of Maryland School of Medicine, University of Maryland, Baltimore, Maryland, USA.; ^2^ Department of Pediatrics, University of Maryland School of Medicine, University of Maryland, Baltimore, Maryland, USA.; ^3^ University of Maryland School of Social Work, University of Maryland, Baltimore, Maryland, USA.; ^4^ University of Maryland Medical Center, University of Maryland, Baltimore, Maryland, USA.; ^5^ University of Maryland School of Nursing, University of Maryland, Baltimore, Maryland, USA.

**Keywords:** advance care planning, graduate medical education, interprofessional education, standardized patients

## Abstract

**Background:**

Improving rates of advance care planning (ACP) and advance directive completion is a recognized goal of health care in the United States. No prior study has examined the efficacy of standardized patient (SP)-based student interprofessional ACP trainings.

**Objectives:**

The present study aims to evaluate an interprofessional approach to ACP education using SP encounters.

**Design:**

We designed a pre–post evaluation of an innovative interprofessional ACP training curriculum using multimodal adult learning techniques to test the effects of completing ACP discussions with SPs. Three surveys (pre-training T1, post-training T2, and post-clinical encounter T3) evaluated student knowledge, Communication Self-Efficacy (CSES), ACP self-efficacy, and interprofessional teamwork (using SPICE-R2).

**Setting/Subjects:**

Students from the schools of medicine, nursing, and social work attended three training modules and two SP encounters focused on ACP.

**Measurements/Results:**

During academic year 2018–2019, 36 students participated in the training at University of Maryland. Results demonstrated statistically significant improvements in ACP self-efficacy, *M*_T1_ = 2.9 (standard deviation [SD]_T1_ = 0.61) compared with *M*_T3_ = 3.9 (SD_T3_ = 0.51), *p* < 0.001, and CSES, *M*_T1_ = 4.6 (SD_T1_ = 1.35) versus *M*_T3_ = 7.3 (SD_T3_ = 0.51), *p* < 0.001, from T1 to T3. There was a medium-to-large improvement in knowledge from an average score of 4.3 (SD = 1.0) at T1 to an average score of 5.5 (SD = 1.4) at T2, *p* = 0.005, *d* = 0.67.

**Conclusions:**

Our interprofessional training module and SP encounter was successful in improving medical, social work, and nursing students' self-reported communication skills and knowledge regarding ACP.

## Introduction

Interprofessional education (IPE) is defined as occasions when individuals from one or more professions learn with, from, or about one another to improve collaboration and the quality of care.^1^ Interprofessional collaboration is associated with enhanced health outcomes for individuals with chronic and complex disease,^2,3^ increased access to and coordination of health services, more appropriate use of health services,^4^ reduced mortality rates,^5^ reduced length of hospital stays,^3,5,6^ and reduced clinical errors.^1,7^

Advance care planning (ACP) is a process of communicating preferences about future care decisions between a patient, their family, and health care providers. This often includes goals-of-care discussions and completion of advance directives (ADs). The ACP reduces unwanted escalation of care,^8^ increases the use of palliative care and hospice services,^9^ and reduces tension between families and health care providers.^10^ Providers and families face challenges when end-of-life care plans are absent.

Implementing ACP has historically encountered multiple barriers, including provider discomfort in discussing end-of-life topics,^11,12^ tensions between the patient and their family members,^13^ misunderstandings of what ACP entails,^14^ lack of formal training in ACP for health care students,^15,16^ and scheduling and other logistical issues.^17,18^ Addressing ACP from an interprofessional approach addresses some of these challenges. Studies have demonstrated that interprofessional care can increase the prevalence of ADs,^19^ help health care students feel more prepared and open to ACP,^20^ increase palliative knowledge and readiness,^21^ and improve patient care.^22^

Standardized patients (SPs), defined as laypersons “who simulate to portray the role of a patient with various health-related conditions,”^23^ have increasingly been integrated into IPE trainings.^24^ This enables health care students to gain experience in treating various health conditions in a safe, controlled setting.^25,26^ The use of SPs has been shown to advance student appreciation and competencies for placing patients' needs and values at the center of interprofessional health care delivery.^27–29^

The SPs can be a particularly powerful learning tool for complex and chronic health care conditions, making them particularly useful for palliative and ACP IPE.^30^ In interprofessional palliative care trainings, simulations have been shown to increase both general and palliative-specific self-efficacy, improve attitudes to team-based learning, improve general preparedness for working with end-of-life patients, and improve attitudes toward palliative care.^31–35^

Although SPs have been shown to be effective in elevating competencies and comfort in ACP among interprofessional health care clinicians in palliative care settings, to the best of our knowledge, no prior study has examined the efficacy of SP-based ACP trainings for interprofessional students. The present study aims at addressing this apparent gap in knowledge by evaluating an interprofessional approach to ACP education of students in medicine, nursing, and social work using SPs.

## Methods

We designed a pre–post evaluation of an innovative interprofessional ACP training curriculum using multimodal adult learning techniques to test the effects of completing ACP discussions with SPs. An educational curriculum dedicated to ACP and interprofessional teamwork, based on the curriculum described in Millstein et al., 2020 was refined by faculty members and specialists from the Schools of Medicine, Social Work, and Nursing. During the 2018–2019 academic year, student participants attended the curriculum, which consisted of three training modules (previously described in Millstein et al., 2020), and two newly developed SP encounters focused on ACP. The training modules were delivered by the interprofessional faculty team, with curricular input from a health care lawyer and attendance by a certified hospital chaplain.

A convenience sample of 1st- and 2nd-year medical students in the Primary Care Track or in the Geriatric Student Interest Group, second (advanced) year clinical social work students in the Aging Specialization or the Health Specialization Tracks, and undergraduate and graduate nursing students were recruited through emails, faculty announcements of the training, and school flyers, with a recruitment goal of 45 students (15 from each profession). All participants were provided written informed consent before participation, and the study received approval from the Institutional Review Board.

### SP encounters

Two SP encounters were developed. The SPs were trained by the interprofessional faculty team. The first encounter demonstrated a patient interested in discussing ACP and open to completing ADs. The second encounter offered increased challenge, as the patient was focused on finding cures for a chronic illness rather than discussing ACP. The second encounter sought to enhance skills by having students negotiate those barriers.

After the delivery of the three training modules, students attended one of two scheduled afternoons. Students were divided into interprofessional teams of two to four and participated in each of the two SP encounters consecutively, followed by team feedback sessions with each SP, and a large group debriefing run by the faculty.

### Participant characteristics

Participants included currently enrolled graduate and undergraduate students pursuing health care careers (Table 1). Thirty-six participants were initially recruited. Age ranged from 20 to 62 years (mean [*M*] = 26.9, standard deviation [SD] = 7.9). Participants were mostly non-Hispanic (*n* = 35, 97.2%), White (*n* = 24, 66.7%), and female (*n* = 27, 75.0%). The academic discipline of participants varied, with 21 (58.3%) studying medicine, 7 (19.4%) nursing, and 8 (22.2%) social work. Twenty-one (58.5%) reported pursuing doctoral degrees, 12 (33.3%) Masters degrees, and 3 (8.3%) Bachelors degrees. The majority of participants indicated that they had never facilitated ACP conversations (*n* = 33, 91.7%) and that they themselves did not have an AD (*n* = 31, 86.1%).

**Table 1.** Sample Characteristics (*n* = 36)

Characteristic	*n* (%)
Age, *M* (SD), year	26.9 (7.6)
Gender	
Female	27 (75.0)
Male	9 (25.0)
Ethnicity	
Hispanic	1 (2.8)
Non-Hispanic	35 (97.2)
Race	
White	24 (66.7)
Black	8 (22.2)
Asian	3 (8.3)
Mixed race or other race	1 (2.8)
Academic discipline	
Medicine	21 (58.3)
Nursing	7 (19.4)
Social work	8 (22.2)
Degree	
Doctorate	21 (58.3)
Master	12 (33.3)
Bachelor	3 (8.3)
Prior experience facilitating advance care planning	
Never	33 (91.7)
One or two times	1 (2.8)
Three or more times	2 (5.6)
Advance directive	
Yes	5 (13.9)
No	31 (86.1)

*M*, mean; SD, standard deviation.

### Data collection

**FIG. 1.**
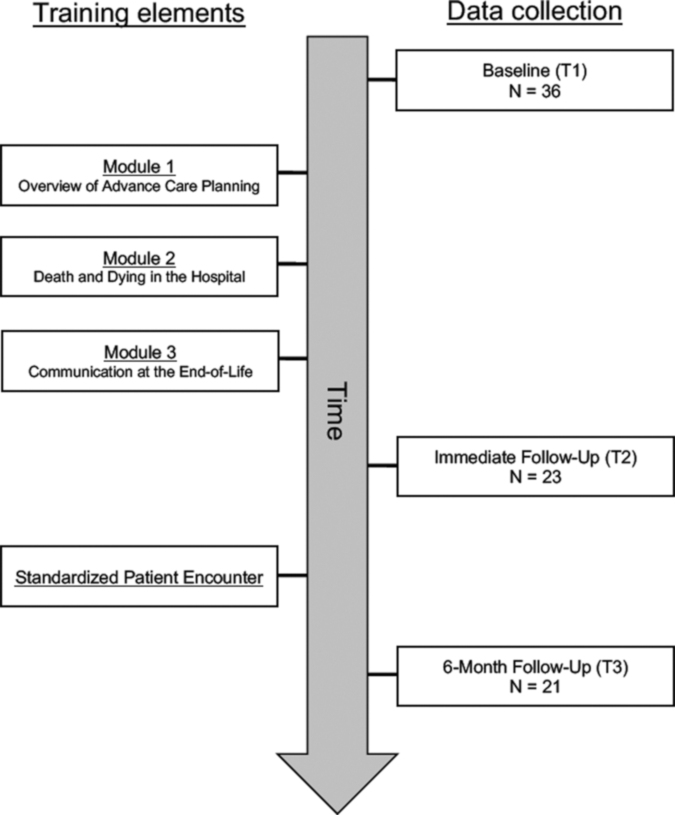
Study flow (*n* = 36).

Data were collected via self-administered Qualtrics surveys from participants at three points: baseline (T1), upon conclusion of Module 3 but before SP encounter (T2), and six months after baseline (T3; Fig. 1). Thirty-six participants completed T1 data collection, 23 participants completed T2 data collection, and 21 participants completed T3 data collection. Thus, the attrition rate from T1 to T2 was 36.1% and the attrition rate from T1 to T3 was 41.6%.

### Measures

The primary outcome measures described next were used in similar interventions previously conducted (Millstein et al., 2020).

#### Primary measures

##### Advance Care Planning Self-Efficacy

Advance Care Planning Self-Efficacy (ACP-SE) was measured using the ACP-SE Scale,^36^ which assesses participants' confidence in facilitating ACP conversations and has been used in similar past research (Millstein et al., 2020; α = 0.94). The ACP-SE contains 17 items scored from 1 (*not at all confident*) to 5 (*very confident*). Items are averaged for a final score (theoretical range = 1–5), with higher scores indicating higher ACP self-efficacy. The ACP-SE demonstrated high internal consistency reliability at T1 (α = 0.93).

##### Communication Self-Efficacy

Communication Self-Efficacy (CSES) was measured through Nørgaard et al.'s^37^ eight-item version of the CSES Scale,^38^ which measures participants' perceptions of their own ability to take part in difficult clinical conversations. Responses are provided on a scale from 1 (*not certain at all*) to 10 (*quite certain*), and then averaged for a final score (possible range = 1–10). Higher average scores indicate higher CSES. Prior research found that this abbreviated version of the CSES demonstrated high internal consistency reliability^20,39^ (α = 0.91–0.93). Internal consistency reliability for the eight-item CSES at T1 was high (α = 0.94).

##### Interprofessional teamwork

We used the Student Perceptions of Interprofessional Clinical Education–Revised 2 (SPICE-R2) scale^40^ to assess interprofessional teamwork. This instrument was developed to evaluate elements of teamwork and collaborative practice among health-affiliated professions participating in an IPE experience.^40–42^ The SPICE-R2 contains 10 items scored on a scale from 1 (*strongly disagree*) to 5 (*strongly agree*). Items are arrayed across three subscales: Interprofessional Teamwork and Team-Based Practice (four items), Roles/Responsibilities for Collaborative Practice (three items), and Patient Outcomes from Collaborative Practice (three items).

We averaged item responses to produce a total score (possible range = 1–5), with higher scores signifying more positive attitudes toward collaborative practice across professions. The SPICE-R2 has demonstrated favorable internal consistency reliability (α = 0.79) in previous studies.^40^ The SPICE-R2 demonstrated similar internal consistency reliability with the current study's full sample at T1 (α = 0.81).

#### Secondary measures

##### Previous training/experience and comfort dealing with difficult conversations

Participants were asked five questions about their previous experience with ACP. Questions exploring participants' specific teaching or training on ACP (“formal teaching/didactics,” “observations,” “another teaching/training experience,” and “none”) and participants' training on responding to patients' emotions (“formal teaching,” “observation,” “both,” “neither”) were asked at T1. Self-rated perceptions of the participant's ability to break bad news (5 = *very good* to 1 = *very poor*), comfort in dealing with a patient's emotions (3 = quite comfortable, 2 = not very comfortable, 1 = uncomfortable), and utilization of a specific plan or strategy when breaking bad news (3 = yes, 2 = somewhat, 1 = no) were asked at T1 and T3.

##### ACP knowledge

Participants were given a seven-question knowledge test at T1 and T2 based on ACP content covered in the modules. Items were scored (0 = incorrect, 1 = correct) by the research team and summed for a final score (possible range = 0–7).

##### Training module evaluation

Each training module was evaluated by participants overall and to assess whether it accomplished the learning objectives using a 5-point response scale (5 = *excellent* to 1 = *poor*).

##### SP simulation experience evaluation

We asked participants to evaluate their SP simulation experiences in three ways. First, participants were asked to rate their SP experience using three items on the T3 survey. The first item asked, “How would you rate the patient simulation experience as a tool for improving your ACP communication skills?” on a five-point scale ranging from “very helpful,” to “very unhelpful.” Next, participants rated their own performance at facilitating ACP communication during the patient simulations using a 10-point scale (0 = *very poor* to 10 = *very strong*). Last, using a free-text response field, participants described their general impressions of the patient simulation experience. Exemplary verbatim quotes from this last item are reported for context.

### Analysis

SAS (Version 9.4) was used for all analyses. We used descriptive statistics to summarize our sample in terms of age, gender, ethnicity, race, academic discipline, academic degree pursued, prior experience facilitating ACP, and whether the student had personally completed an AD for themself. Descriptive statistics were used to report participants' scores on the ACP-SE, CSES, and SPICE-R2 primary measures. All descriptive statistics are presented as appropriate to the level of measure (i.e., *n* [%] for categorical data, *M* [SD] for continuous data). Assumptions for all inferential analyses were verified before analysis. All analyses were two-sided and used a statistical significance level of *p* = 0.05.

#### Primary analyses

We performed paired-samples *t* tests to assess mean differences across the three time points: (1) T1 to T2, (2) T2 to T3, and (3) T1 to T3. To facilitate the comparison of results across study measures, Cohen's *d* effect size was calculated using G*Power (Version 3.1) for all paired samples *t*-test results. We used the effect size descriptors (0.01 = very small, 0.20 = small, 0.50 = medium, 0.80 = large, 1.20 = very large, 2.00 = huge) proposed by Cohen^43^ and Sawilowsky^44^ to interpret the magnitude of effect. Positive and negative effect size values are reported to indicate beneficial effects (positive values) and detrimental effects (negative values).

#### Secondary analyses

Descriptive statistics were presented for all secondary measures as appropriate per each variable's level of measurement. We performed nonparametric Wilcoxon signed-ranks tests for the measures on previous training/experience and comfort dealing with difficult conversations administered at T1 and again at T3. Finally, we conducted a paired samples *t* test to assess changes in ACP knowledge. Effect sizes for these repeated measures analyses (Wilcoxon signed-ranks test [*d_z_*] and paired-samples *t* test [Cohen's *d*]) were calculated and interpreted as referenced earlier. Pairwise deletion was used.

## Results

### Primary results

#### T1 to T2

Results showed that outcomes initially worsened from T1 to T2 for participants' ACP self-efficacy (*M*_T1_ = 3.0; SD_T1_ = 0.54 compared with *M*_T2_ = 2.3; SD_T2_ = 0.51, *p* < 0.001, *d* = −0.91) and CSES (*M*_T1_ = 4.7; SD_T1_ = 1.1 relative to *M*_T2_ = 3.8; SD_T2_ = 1.4, *p* = 0.006, *d* = −0.64). Interprofessional teamwork improved slightly from T1 to T2 (*M*_T1_ = 4.0; SD_T1_ = 0.45 compared with *M*_T2_ = 4.3; SD_T2_ = 0.51, *p* = 0.001, *d* = 0.79).

#### T2 to T3

Statistically significant improvements were observed from T2 to T3 for ACP self-efficacy (*M*_T2_ = 2.4; SD_T2_ = 0.51 compared with *M*_T3_ = 3.8; SD_T3_ = 0.50, *p* < 0.001, *d* = 2.8) and CSES (*M*_T2_ = 3.9; SD_T2_ = 1.5 vs. *M*_T3_ = 7.3; SD_T3_ = 1.1, *p* < 0.001, *d* = 2.6). On average, participants scored 1.46 points (standard error [SE] = 0.19) higher on ACP self-efficacy and 3.48 points (SE = 0.56) higher on CSES. No change was detected for interprofessional teamwork (*M*_T2_ = 4.3; SD_T2_ = 0.40 relative to *M*_T3_ = 4.1; SD_T3_ = 0.88, *p* = 0.264, *d* = 0.3).

#### T1 to T3

**FIG. 2.**
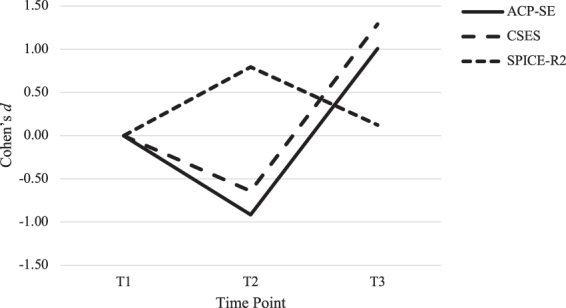
Effect sizes of the changes in primary measures across time points. ACP, advance care planning; ACP-SE, Advance Care Planning Self-Efficacy; CSES, Communication Self-Efficacy; SPICE-R2, Student Perceptions of Interprofessional Clinical Education–Revised 2.

Relative to baseline, the assessment of longer term outcomes found improvements in ACP self-efficacy (*M*_T1_ = 2.9; SD_T1_ = 0.61 compared with *M*_T3_ = 3.9; SD_T3_ = 0.51, *p* < 0.001) and CSES (*M*_T1_ = 4.6; SD_T1_ = 1.35 vs. *M*_T3_ = 7.3; SD_T3_ = 0.51, *p* < 0.001) from T1 to T3. On average, participants' scores regarding ACP self-efficacy improved by 0.97 points (SE = 0.22). Participants' scores regarding CSES improved by an average of 2.79 points (SE = 0.47). Effect size estimates revealed a large-to-very large effect for ACP self-efficacy (*d* = 1.01) and a very large effect in CSES (*d* = 1.29). Effect size conversions relative to the T1 baseline are illustrated in Figure 2. A difference was not observed in interprofessional teamwork from T1 to T3 (*M*_T1_ = 4.0; SD_T1_ = 0.50 compared with *M*_T3_ = 4.1; SD_T3_ = 0.87, *p* = 0.590, *d* = 0.12).

### Secondary results

#### Previous training/experience and comfort dealing with difficult conversations

The T1 results indicated that most participants reported having had no previous teaching or training regarding ACP (69.4%). Across the four response options regarding previous training on responding to patients' emotions, participants were equally split between indicating that they had (1) observational experience, and (2) had neither formal training nor observational experience (36.1% for each).

Results of the Wilcoxon signed-ranks test revealed a statistically significant difference from T1 to T3 in the ability to break bad news, *T*(*n* = 22) = 45.5, *p* < 0.001. The median response at T1 was “fair” compared with “good” at T3. Utilizing a consistent strategy for breaking bad news also improved slightly from T1 to T3, with the median response at T1 being the midpoint between having “no” strategy and “somewhat” of a strategy compared with a median response of “somewhat” at T3, *T*(*n* = 22) = 33.0, *p* = 0.001. There was no observed statistical difference in comfort in dealing with patients' emotions, *T*(*n* = 22) = 3.5, *p* < 0.688.

#### ACP knowledge

There was a medium-to-large improvement in knowledge from an average score of 4.3 (SD = 1.0) at T1 to an average score of 5.5 (SD = 1.4) at T2, *p* = 0.005, *d* = 0.67.

#### Training module evaluation

Participants generally rated the training modules positively. Overall, module quality was reported as “very good” or “excellent” by 51.7%, 71.4%, and 86.6% of participants in Module 1, 2, and 3, respectively. The assessment of meeting learning objectives was similarly reported as “very good” or “excellent” by 62%, 78.6%, and 80% for Module 1, 2, and 3, respectively.

#### Standardized patient simulation experience evaluation

Based on T3 data, 91.7% of participants indicated that the SP simulation experience was “very helpful,” with the remaining 8.3% indicating “helpful.” All participants rated their personal performance at facilitating ACP during the SP activity at least a five or above (*M* = 7.3, SD = 1.3; possible range from 0 = *very poor* to 10 = *very strong*). Qualitative impressions were consistently positive (Table 2). Participants also provided recommendations for future improvements, including suggestions for scheduling, greater interdisciplinary involvement (e.g., to ensure that one representative from each discipline is always present), and to increase SP simulation experiences.

**Table 2.** Exemplary Comments Regarding Standardized Patient Simulation Experience

• I would definitely recommend patient simulations for future cohorts. It was a great opportunity to put into practice everything we learned in the three modules. I would recommend keeping one difficult patient and one easy patient, just like the simulation was. The difficult patient scenario was hard and a little uncomfortable but it was great practice for real life!
• This was the most valuable experience from the entire ACP series.
• I would emphatically recommend patient simulation experiences to future students. I genuinely believe that they are the best way to learn and to discover how to apply this material.

ACP, advance care planning.

## Discussion

Our interprofessional training module and SP encounter was successful in improving medical, social work, and nursing students' self-reported communication skills and knowledge regarding ACP. Statistically significant improvement with large effect sizes were seen in key study outcomes at six months (T3), with the exception of interprofessional teamwork. To our knowledge, this is the first study to combine IPE, SP, and ACP. The improvement in outcomes from T2 to T3 suggests that the SP experience was an exceptionally valuable applied component of the curriculum.

Data collection immediately after education modules (T2) reported lower scores than baseline (T1) and six-month follow-up (T3). These findings were previously reported (Millstein et al., 2020) and are replicated in this study. The authors hypothesize that this finding is due to students initially overestimating their own ability to communicate about ACP issues effectively and subsequently recognizing the high complexity of ACP through the education modules and role play activities. This may be further explained by the fact that students were novice in their clinical experience, education, and exposure to ACP.

This finding is consistent with the Dunning/Kruger effect in which unskilled novices overestimate their self-efficacy and skills followed by a drop in confidence with exposure to the task.^45^ Future research to improve ACP skills should evaluate whether this self-assessment “correction” holds with other health care providers working with this population (i.e., patient navigators, health care chaplains, nursing assistants, primary care providers, skilled nursing administrators), and if so, researchers and clinicians should account for this correction when developing trainings and evaluating outcomes.

Our findings should be considered within the context of the study's limitations. Our limited sample size may have contributed to null findings. In addition, the lack of a control group does not allow for us to make definitive statements about the impact of training on learning due to the possible influence of external experiences.

Future steps include the evaluation of similar interprofessional curricula on ACP education. Based on student feedback and faculty observations, key areas to target include continued emphasis on modeling, role-play, use of SPs, clinical reflection, and interprofessional student team practice. Future study is needed to explore how different disciplines perceive, and benefit from, these various learning activities. The use of SPs allows for less variability in student experience but may present a financial barrier. Faculty or other trained volunteers playing the role of the patient may alleviate this issue. Further, the lack of improvement on students' ability to respond to patient emotions suggests that training modules and role-plays could be modified to better address patient affect.

Despite the implementation challenges of IPE programs in university settings,^46^ our team was able to successfully implement a second year of a well-attended, high-impact training on ACP for social work, nursing, and medical students. Although our evaluation of training outcomes suggests that learners ultimately benefited from the experience, future research is needed to demonstrate improvement in real-world clinical settings.

## Abbreviations Used

ACP: advance care planning
ACP-SE: Advance Care Planning Self-Efficacy
ADs: advance directives
CSES: Communication Self-Efficacy
IPE: interprofessional education
*M*: mean
SD: standard deviation
SE: standard error
SP: standardized patient
SPICE-R2: Student Perceptions of Interprofessional Clinical Education–Revised 2
UMB: University of Maryland, Baltimore
